# scTPC: a novel semisupervised deep clustering model for scRNA-seq data

**DOI:** 10.1093/bioinformatics/btae293

**Published:** 2024-04-29

**Authors:** Yushan Qiu, Lingfei Yang, Hao Jiang, Quan Zou

**Affiliations:** School of Mathematical Sciences, Shenzhen University, Shenzhen, Guangdong 518000, China; School of Mathematical Sciences, Shenzhen University, Shenzhen, Guangdong 518000, China; School of Mathematics, Renmin University of China, Haidian District, Beijing 100872, China; Institute of Fundamental and Frontier Sciences, University of Electronic Science and Technology of China, Chengdu 610056, China

## Abstract

**Motivation:**

Continuous advancements in single-cell RNA sequencing (scRNA-seq) technology have enabled researchers to further explore the study of cell heterogeneity, trajectory inference, identification of rare cell types, and neurology. Accurate scRNA-seq data clustering is crucial in single-cell sequencing data analysis. However, the high dimensionality, sparsity, and presence of “false” zero values in the data can pose challenges to clustering. Furthermore, current unsupervised clustering algorithms have not effectively leveraged prior biological knowledge, making cell clustering even more challenging.

**Results:**

This study investigates a semisupervised clustering model called scTPC, which integrates the triplet constraint, pairwise constraint, and cross-entropy constraint based on deep learning. Specifically, the model begins by pretraining a denoising autoencoder based on a zero-inflated negative binomial distribution. Deep clustering is then performed in the learned latent feature space using triplet constraints and pairwise constraints generated from partial labeled cells. Finally, to address imbalanced cell-type datasets, a weighted cross-entropy loss is introduced to optimize the model. A series of experimental results on 10 real scRNA-seq datasets and five simulated datasets demonstrate that scTPC achieves accurate clustering with a well-designed framework.

**Availability and implementation:**

scTPC is a Python-based algorithm, and the code is available from https://github.com/LF-Yang/Code or https://zenodo.org/records/10951780.

## 1 Introduction

During the past decade, there have been remarkable advancements in single-cell RNA sequencing (scRNA-seq) technology, which it is now widely applied in various contexts within the biomedical domain. The inception of the scRNA-seq dates to 2009, at its initial introduction (Tang *et al.* 2009). This is an advanced method for analyzing gene expression, characterized by its ability to investigate genetic activity at the single-cell level. Unlike conventional transcriptomic sequencing, this innovative approach delineates distinct gene expression patterns of individual cells, thereby capturing the diverse cellular heterogeneity of the overall population. Thus, clustering serves as the primary stage of scRNA-seq data analysis. The efficacy of the clustering process significantly influences subsequent downstream analyses, including cell type identification, gene expression analysis, and trajectory inference. Hence, the precise clustering of scRNA-seq data is of paramount importance, as it lays the foundation for the accurate interpretation of results and the extraction of meaningful insights.

The challenges encountered in scRNA-seq data clustering research stem primarily from the inherent nature of the single-cell sequencing data itself. First, the data are characterized by their high-dimensional structure, where each dimension corresponds to a specific gene expression quantity. As the number of dimensions increases, the complexity of the data increases, creating the predicament known as the “curse of dimensionality” (Clark *et al.* 2019). Second, the high sparsity of the data poses another obstacle (Eling *et al.* 2019). Since not all genes within cells exhibit expression, there is a considerable proportion of unexpressed genes and an abundance of zero values within the scRNA-seq data. Finally, the occurrence of “dropout” events further complicates the analysis (Hedlund and Deng 2018). Some of the zero values represent the genuine biological absence of gene expression, whereas others result from deficiencies in sequencing technology, leading to incomplete data capture. Consequently, there exists a certain probability of losing gene expression data, resulting in “false” zero values.

Currently, numerous clustering methods have been proposed for scRNA-seq analysis. Most of these methods are based on traditional dimensionality reduction and clustering algorithms (Todorov *et al.* 2018). The simplest approach involves using techniques like PCA (Yang *et al.* 2004) and nonnegative matrix factorization (NMF) (Lee and Seung 1999) for dimensionality reduction, followed by K-means (Wang *et al.* 2017) clustering to obtain the clustering results. In addition, similarity-based clustering methods construct cell-to-cell distance metrics to accommodate the characteristics of the data. Currently, the most classical and commonly used methods include SC3 (Kiselev *et al.* 2017), Seurat (Butler *et al.* 2018), and SIMLR (Wang *et al.* 2018a). These methods utilize simple linear dimensionality reduction techniques to directly cluster the similarity or distance learned from the original data or the reduced matrix. However, this approach can lead to inaccurate predictions for noisy and high-dimensional data and fails to capture nonlinear structures within the data. Moreover, treating dimensionality reduction and clustering as independent operations can result in inaccuracies in the fidelity of the final results. Due to the nonnegativity of gene expression matrix values in scRNA-seq data, NMF exhibits good adaptability to nonnegative data and can better capture features. DRjCC (Wu and Ma 2020) and jSRC (Wu *et al.* 2021) are NMF-based joint learning algorithms that combine sparse representation and clustering. However, they utilize only feature sparsity and lack interpretability. SLNMF (Wu and Ma 2023) is a NMF algorithm based on network structure learning. Initially, a similarity network of cells is constructed, and then by leveraging the topological structure of the intercellular network, latent features of cells are extracted. By preserving the structural information of the network to learn the latent features of cells. In addition, NIC (Wu *et al.* 2022) is another network-based single-cell data analysis method within the NMF framework, which also leverages the learning of similarity networks among cells to enhance feature extraction.

In the field of computational biology, there has been a remarkable application of deep learning methods that have exhibited exceptional performance. Given the presence of nonlinear relationships among cells in scRNA-seq data, deep learning techniques have emerged as a valuable approach for extracting nonlinear cell features. By seamlessly incorporating deep neural network models into single-cell clustering, more efficient feature extraction and clustering of scRNA-seq data can be achieved. A notable contribution in this area is the work of Eraslan *et al.* (2019) who introduced deep count autoencoder (DCA) network. This model effectively denoises the scRNA-seq data and learns informative features in an unsupervised manner. Based on this finding, Tian *et al.* (2019) combine the DCA model with the DEC (Xie *et al.* 2016) clustering algorithm, resulting in a deep embedding clustering approach known as scDeepCluster. This method encompasses feature learning and incorporates clustering tasks based on single-cell models. Additionally, scziDesk (Chen *et al.* 2020) integrates data modeling, dimensionality reduction, and cell clustering through iterative updates of the data denoising and clustering processes. By employing neighborhood contrastive loss and auxiliary mask estimation tasks, scNAME (Wan *et al.* 2022) fully explores the correlations between features and the similarities among cells. These innovative methodologies enhance our ability to unlock new insights from scRNA-seq data.

The clustering methods mentioned above are classified as unsupervised algorithms, because they do not take advantage of prior information, which can impact the quality of clustering results. To address this limitation, researchers are now exploring semisupervised algorithms that harness limited labeled information to guide the clustering process and enhance clustering outcomes. For example, scDCC (Tian *et al.* 2021) converts prior knowledge into pairwise constraints and incorporates them as additional terms in the loss function of the deep learning model. Thus, scDCC enables the model to learn more accurate latent representations, leading to improved clustering results. Another approach, scSSA (Zhao *et al.* 2022), combines semisupervised autoencoders and FastICA for dimensionality reduction, followed by the use of Gaussian mixture models with the BIC index for clustering. This methodology effectively leverages labeled information to inform dimensionality reduction and clustering. Similarly, scSemiAAE (Wang *et al.* 2023) introduces an autoencoder architecture based on the zero-inflated negative binomial (ZINB) loss. By integrating adversarial training and a semisupervised module within the latent space, scSemiAAE effectively takes advantage of labeled information to enhance clustering results. These semisupervised algorithms represent valuable advancements in the field, enabling the incorporation of prior knowledge and labeled information to achieve more accurate and informative clustering outcomes.

Considering the enormous amount of sequencing data available today, coupled with the identification of marker genes (Clark *et al.* 2019) for distinct cell types and the existence of labeled datasets, it has become increasingly crucial to explore methods that capitalize on prior biological knowledge and leverage labeled information to enhance the performance of clustering algorithms. This need for tailored approaches that suit the characteristics of sequencing data has motivated our research.

**Figure 1.**
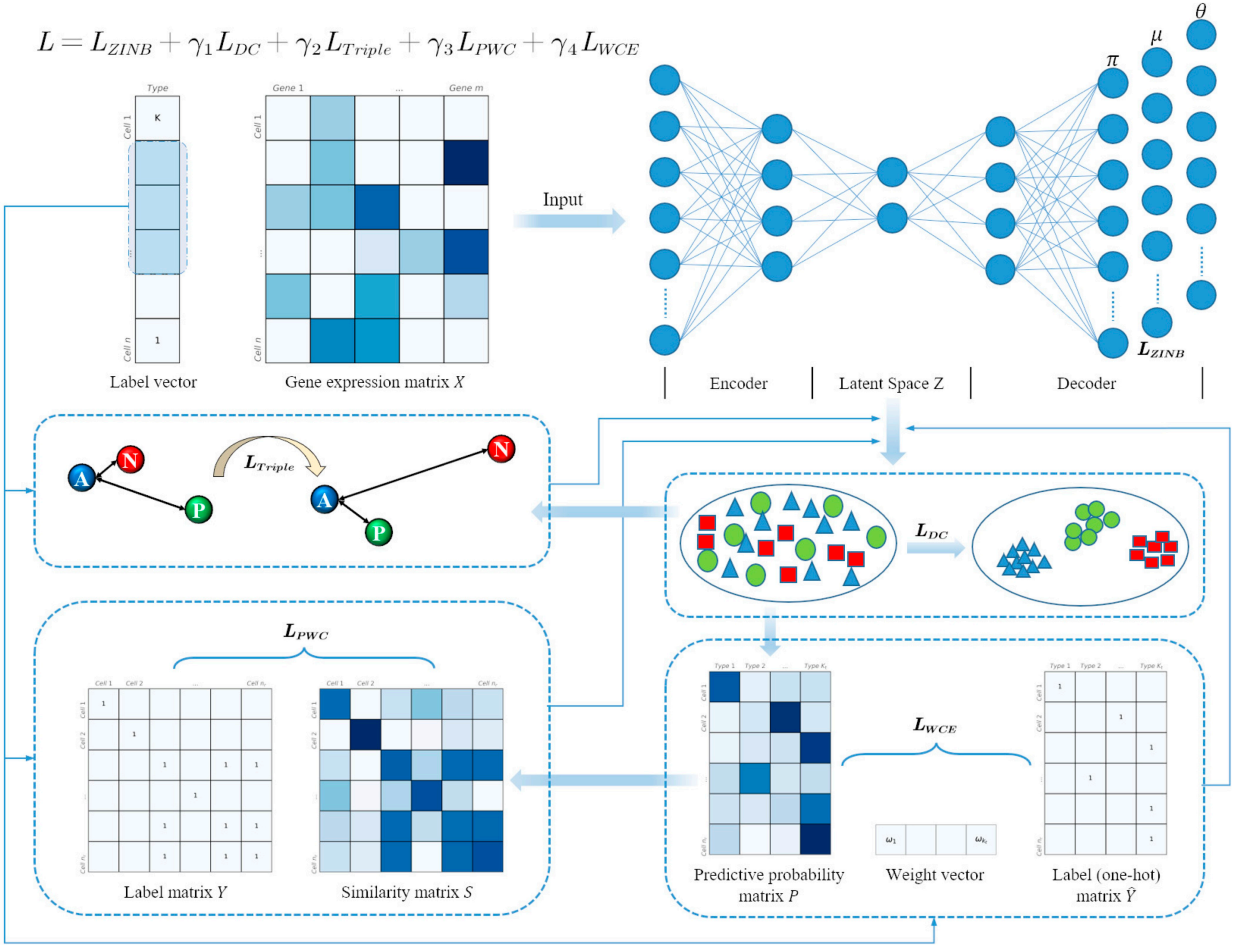
Overview of scTPC. A deep denoising autoencoder based on ZINB is constructed, with symmetrical structures for both the encoder and the decoder. The scRNA-seq data are used as input, and the outputs are three sets of parameters: dropout rate, mean value, and dispersion value. Deep clustering is performed on the embedded points in the latent space. The label information is integrated to generate triplet and pairwise constraints, and weighted cross-entropy is introduced to balance the dataset.

Taking inspiration from scDeepCluster, the deep learning-based clustering method, we developed a clustering approach called scTPC. This novel method combines a ZINB distribution denoising autoencoder with the integration of labeled information. Our approach assumes that the denoised scRNA-seq data obtained through the autoencoder can be accurately described by the ZINB model in terms of the overall probability structure. The scTPC method utilizes deep clustering in a low-dimensional feature space and incorporates triplet generation based on known labels and pairwise constraints, to guide the clustering process. To address the issue of data imbalance in the dataset, we also introduced a weighted cross-entropy optimization model. The architecture of the scTPC is depicted in Fig. 1. Using this innovative approach, we aim to improve the accuracy and effectiveness of clustering in scRNA-seq data by capitalizing on both the denoising capabilities of the autoencoder and the valuable label information available. This method has great potential for advancing our understanding of cell types and their characteristics within complex biological systems.

## 2 Materials and methods

### 2.1 ZINB denoising autoencoder

The scRNA-seq data are characterized by high sparsity and the occurrence of “dropout” events. To effectively account for these characteristics, we employ a denoising autocoder based on ZINB. This distribution not only captures the dispersion and sparsity of the data but also provides a comprehensive representation of the overall data distribution. Similar to many other deep learning-based methods for single-cell clustering, we used a ZINB-based denoising autocoder to reconstruct the scRNA-seq data. The ZINB distribution is a combination of a zero component and a negative binomial distribution, which is described by the following formula:

(1) $$P_{\text{NB}}\left(X\;|\;\mu ,\theta \right)=\frac{\Gamma \left(X\;+\;\theta \right)}{\Gamma \left(X\;+\;1\right)\Gamma \left(\theta \right)}{\left(\frac{\mu }{\mu \;+\;\theta }\right)}^{X}{\left(\frac{\theta }{\mu \;+\;\theta }\right)}^{\theta },$$

(2) $$P_{\text{ZINB}}\left(X\;|\;\pi ,\mu ,\theta \right)=\pi \delta_{0}\left(X\right)\;+\;\left(1\;-\;\pi \right)P_{\text{NB}}\left(X\;|\;\mu ,\theta \right).$$

Here, *X* denotes the count matrix, with $X_{ij}$ representing the expression value of the *j*-th gene in the *i*-th cell. Within the ZINB distribution, π, μ, and θ represent the probability of dropout, the mean, and the dispersion, respectively.

Regarding the reconstruction loss, the loss function employed is the negative log-likelihood of the ZINB distribution which can be expressed as follows:

(3) $$L_{\text{ZINB}}=-\text{log}\;P_{\text{ZINB}}\left(X\;|\;\pi ,\mu ,\theta \right).$$

### 2.2 Deep clustering

After training the autoencoder, we obtained the latent feature representation in low-dimensional space. After which we can engage in deep clustering within latent space. In contrast to the conventional approach of involving the direct application of the K-means algorithm in the latent space to obtain clustering results, we employed a weighted K-means method, similar to scziDesk (Chen *et al.* 2020), to obtain cluster centers. The objective function can be expressed as follows:

(4) $$L_{\text{Soft}\_K}=\sum_{i}^{n}\sum_{j}^{K}\omega_{ij}{\left\|z_{i}\;-\;\varphi_{j}\right\|}^{2}.$$

Here $z_{i}$ represents the latent expression of the *i*th sample, and $\varphi_{j}$ denotes the *j*th cluster center. Meanwhile, $\omega_{ij}$ symbolizes the weight of sample *i* belonging to the *j*th cluster. Similar to scziDesk (Chen *et al.* 2020), we employ the Gaussian kernel function to measure ω, accompanied by dilation processing to accelerate the convergence rate (Vlasblom and Wodak 2009).

Formally, deep clustering (Xie *et al.* 2016) is defined as the Kullback–Leibler (KL) divergence (Guo *et al.* 2017) between the distributions of *P* and *Q*. Here, *Q* represents the soft label distribution, measured using Student’s *t*-distribution (Van der Maaten and Hinton 2008), while *P* denotes the *Q*-derived target distribution. In deep clustering, *Q* distribution is used for clustering in the low-dimensional embedding space, guiding the network update through the KL divergence concerning the *Q*-dependent target distribution *P* (Nigam and Ghani 2000). Then the clustering loss term is defined as follows:

(5) $$L_{\text{KL}}=\sum_{i}^{n}\sum_{j}^{K}p_{ij}\;\text{log}\;\frac{p_{ij}}{q_{ij}}.$$

Here, $q_{ij}$ represents the soft-assigned label of point $z_{i}$, defined as the similarity between $z_{i}$ and $\varphi_{j}$ (Van Der Maaten 2009). The distribution *P* is computed based on *Q*, and minimizing KL divergence $L_{\text{KL}}$ in each iteration encourages *Q* to gradually approach the target distribution *P*.

Consequently, the integration of $L_{\text{Soft}\_K}$ and $L_{\text{KL}}$ results in the following clustering loss.

(6) $$L_{\text{DC}}=L_{\text{Soft}\_K}\;+\;L_{\text{KL}}.$$

### 2.3 Triplet constraint

To achieve enhanced clustering results, triplet constraints are introduced in the field of image processing, allowing a more precise discrimination of details (Schroff *et al.* 2015). The fundamental principle behind triplet constraints is to learn a well-structured embedding space where similar samples are close to each other, aiding in determining whether they belong to the same category. The goal of single-cell clustering is to group cells with similar expression patterns together, effectively distinguishing cells of different classes. In view of these, the concept of triplet constraints aligns well with the objective of single-cell clustering, which involves determining whether two samples belong to the same category. Therefore, triplet constraints are naturally applied in our model.

The model is trained by learning triplets that differentiate instances of the same class from those of distinct classes. Specifically, triplet construction involves random sampling from a labeled cell dataset, which can be a known labeled cell dataset or one generated using the Garnett (Pliner *et al.* 2019) scoring technique. The triplet comprises an anchor cell, a positive cell, and a negative cell. Anchor and positive cells belong to the same cell type, whereas the negative cell belongs to a different cell type.

The objective of triplet loss is to minimize the distance between the anchor and positive cells, while maximizing the distance between the anchor and negative cells. Therefore, our aim is to minimize the intraclass distance and maximize the interclass distance.

(7) $$\begin{array}{c} \begin{array}{c} d\left(q_{A},q_{P}\right)\;+\;\alpha \;<\;d\left(q_{A},q_{N}\right)\\ \forall \left(A,N,P\right)\in T \end{array} \end{array}.$$

Here, α serves as the interval parameter, further accentuating the distinction between the anchor-positive pair and the anchor-negative pair. The set *T* denotes all feasible triplets within the dataset. Throughout the deep clustering process, the clustering vector can be derived, with the distance between the anchor cell and the positive cell defined as follows:

(8) $$d\left(q_{A},q_{P}\right)=\sum q_{A}\cdot q_{P}.$$

The triplet constraint loss can be defined as follows:

(9) $$L_{\text{Triple}}=\sum_{\left(A,N,P\right)\in T}\max\left(d\left(q_{A},q_{P}\right)\;-\;d\left(q_{A},q_{N}\right)\;+\;\alpha ,0\right).$$

### 2.4 Pairwise constraints

The deep clustering layer incorporates label information, which guides the data clustering process. However, the guidance provided solely at the clustering level overlooks the pairwise constraints between cells (Chen *et al.* 2021). Typically, two types of constraints are defined: Must-link and Cannot-link (Basu *et al.* 2008). Must-link constraint signifies beforehand that two cells are of the same cell type, whereas the Cannot-link constraint signifies beforehand that two corresponding cells belong to different cell types.

Paired Must-link and Cannot-link constraints are commonly employed in semisupervised algorithms to steer the clustering process (van Engelen and Hoos 2020). Hence, it is advantageous to incorporate these pairwise constraints while clustering based on labeled datasets. Initially, the discriminant probability vector *p* is utilized to compute the similarity matrix *S*, which can be calculated using the following expression:

(10) $$S_{ij}=\frac{{\left(p_{i}\right)}^{T}p_{j}}{\left\|p_{i}\right\|\left\|p_{j}\right\|},$$

where ‖·‖ represents the $L_{2}$ norm. By leveraging the label information, the label matrix *Y* can be constructed, which describes the Must-link and Cannot-link constraint pairs based on the data provided. Specifically, *Y* can be defined as the following:

(11) $$\begin{array}{c} Y_{ij}=\{\begin{array}{c} 1,y_{i}=y_{j}\\ 0,y_{i}\neq y_{j} \end{array} \end{array}.$$

It is desirable to ensure that samples with the same label are in proximity to each other, whereas samples with different labels are distanced from each other. Consequently, the pairwise constraint loss is formulated as follows:

(12) $$L_{\text{PWC}}=\;-\;\sum_{i}^{n_{r}}\sum_{j}^{n_{r}}\left[Y_{ij}\;\text{log}\;S_{ij}\;+\;\left(1\;-\;Y_{ij}\right)\;\text{log}\;\left(1\;-\;S_{ij}\right)\right].$$

### 2.5 Balancing dataset

Considering the imbalance in the sample distribution across different categories in the dataset, where some categories have numerous samples while others have a significantly smaller count, it is crucial to address the resulting bias in the model. This bias often results in poor prediction performance, particularly for categories with limited samples. These categories are more likely to be assigned erroneously to the class that contains a larger number of samples (Kim *et al.* 2021). To mitigate this issue, a weighted cross-entropy loss is introduced, thereby facilitating additional model optimization.

(13) $$L_{\text{WCE}}=\;-\;\frac{1}{n_{r}}\sum_{i=1}^{n_{r}}\sum_{j=1}^{K_{t}}{\hat{\omega }}_{j}{\hat{y}}_{ij}\;\text{log}\;p_{ij},$$

where ${\hat{\omega }}_{j}$ denotes the weight assigned to the *j*th category, ${\hat{y}}_{ij}$ indicates whether the *i*th sample belongs to the *j*th class, and $p_{ij}$ represents the predicted probability of the *i*th sample belonging to the *j*th class. To ensure that the model places greater emphasis on categories with limited samples during training, we assign them higher weights. This deliberate weighting is beneficial to enhance the accuracy of clustering outcomes.

### 2.6 Model training strategy

Our approach comprises five key components: a ZINB-based denoising autoencoder, deep clustering, triplet constraints, pairwise constraints, and a balanced dataset component. Consequently, the holistic objective function of the model can be expressed as follows:

(14) $$L=L_{\text{ZINB}}\;+\;\gamma_{1}L_{\text{DC}}\;+\;\gamma_{2}L_{\text{Triple}}\;+\;\gamma_{3}L_{\text{PWC}}\;+\;\gamma_{4}L_{\text{WCE}}.$$

Here, $\gamma_{1}$, $\gamma_{2}$, $\gamma_{3}$, and $\gamma_{4}$ denote the coefficients governing the relative importance of the different losses. To implement the model training strategy, $L_{\text{ZINB}}$ for 300 epochs were pretrained as the starting point. Subsequently, we proceeded with training the complete model *L* until the assignments of cluster labels reached a state of stability. The framework of the method we investigated is illustrated in Fig. 1.

scTPC uses a deep autoencoder based on the ZINB distribution. The network consists of five layers with connected node sizes of 256, 128, 32, 128, and 256, respectively. The rectified linear unit ReLU() activation function is employed. The batch size for training was set to 256. We used the AdaDelta optimizer with the learning rate and rho parameters set to 1.0 and 0.9, respectively.

Our method has six hyperparameters. *K* (number of clusters) is set to the true number of cell types in the dataset because it is known. α (margin parameter) is set to a default value of 0.1. $\gamma_{1}$, $\gamma_{2}$, $\gamma_{3}$ and $\gamma_{4}$ are determined through grid search to determine their optimal values (results are summarized in the Supplementary File).

## 3 Results and analysis

In this section, we discuss the clustering impact of our model on scRNA-seq data, and compare the clustering outcomes with those obtained from several prominent methods on authentic datasets. In order to appraise the clustering results, we employ four extensively embraced metrics: Normalized Mutual Information (NMI) (Strehl and Ghosh 2002), Adjusted Rand Index (ARI) (Hubert and Arabie 1985), Adjusted Mutual Information (AMI) (Vinh *et al.* 2010), and Accuracy of Clustering (ACC) (see Supplementary File).

### 3.1 Datasets and comparison methods

We applied scTPC to 10 datasets sourced from various species and organs acquired through multiple sequencing platforms. The number of cells in these datasets varied from 2717 to 12 089, encompassing cell types ranging from 2 to 46. Table 1 provides a summary of these real scRNA-seq datasets.

**Table 1.** Summary of the real scRNA-seq datasets.

Dataset	Platform	Cells	Genes	Types	Refs
Mouse_bladder_cell	Microwellseq	2746	20 670	16	(Han *et al.* 2018)
Mouse_ES_cell	Droplet barcoding	2717	24 175	4	(Klein *et al.* 2015)
Worm_neuron_cell	sciRNAseq	4186	13 488	10	(Cao *et al.* 2017)
10X_PBMC	10X	4271	16 653	8	(Zheng *et al.* 2017)
Young	10X	5685	33 685	11	(Young *et al.* 2018)
Plasschaert	Indrop	6977	28 205	8	(Plasschaert *et al.* 2018)
Wang_Lung	10X	9519	14 561	2	(Wang *et al.* 2018b)
Qx_Spleen	10X	9552	23 341	5	(Schaum *et al.* 2018)
Qx_Trachea	10X	11269	23 341	5	(Schaum *et al.* 2018)
Chen	Drop_seq	12089	23 284	46	(Chen *et al.* 2017)

For comparison, we have chosen twelve distinct methods: SC3 (Kiselev *et al.* 2017), Seurat (Butler *et al.* 2018), SIMLR (Wang *et al.* 2018a), scDeepCluster (Tian *et al.* 2019), scziDesk (Chen *et al.* 2020), scDCC (Tian *et al.* 2021), scSemiAAE (Wang *et al.* 2023), scSSA (Zhao *et al.* 2022), DRJCC (Wu and Ma 2020), jSRC (Wu *et al.* 2021), PCA+K-means, and NMF+K-means. This selection encompasses classical single-cell clustering approaches, deep learning-based methodologies, matrix factorization-based techniques, and semisupervised methods for single-cell clustering (The methods are summarized in the Supplementary File).

### 3.2 Simulation experiment

**Figure 2.**
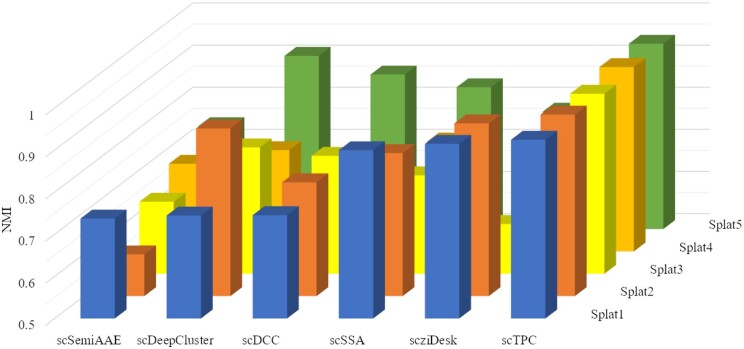
The comparison of NMI value of our model and the deep learning and semisupervised clustering methods on the five simulated datasets.

We initially generated five simulated datasets using the Splatter (Zappia *et al.* 2017), with detailed information summarized in Table 2. Given that the work explores semisupervised clustering methods based on deep learning, thus, we conducted clustering performance comparisons on these five simulated datasets using deep learning-based and semisupervised clustering methods, i.e. scDeepCluster (Tian *et al.* 2019), scziDesk (Chen *et al.* 2020), scDCC (Tian *et al.* 2021), scSemiAAE (Wang *et al.* 2023), scSSA (Zhao *et al.* 2022). The NMI results are showed in Fig. 2 (ARI, ACC, and AMI can be found in the Supplementary File).

**Table 2.** Summary of the simulated datasets.

Dataset	Genes	Cells	Clusters	group.prob
Splat1	10 000	2000	4	c(0.05,0.1,0.2,0.65)
Splat2	10 000	5000	4	c(0.05,0.1,0.2,0.65)
Splat3	10 000	10000	4	c(0.05,0.1,0.2,0.65)
Splat4	10 000	2000	6	c(0.02, 0.03, 0.2, 0.65,0.03,0.07)
Splat5	10 000	2000	10	c(0.02,0.02,0.03,0.05,0.08,0.1,0.1,0.15,0.15,0.3)

Overall, across the five simulated datasets, scTPC is consistently superior to the state-of-the-art methods. In addition, datasets Splat1, Splat2, and Splat3 differ in terms of the number of cells and our model outperforms the other methods for these datasets with different number of cells. Besides, datasets Splat1, Splat4, and Splat5 differ in the number of clusters and scTPC performs better than the state-of-the-art methods on these datasets with different number of clusters. Our method demonstrates robust clustering performance across all datasets, indicating its adaptability to different data distributions in the simulated experiments.

### 3.3 Visualization and comparison

**Figure 3.**
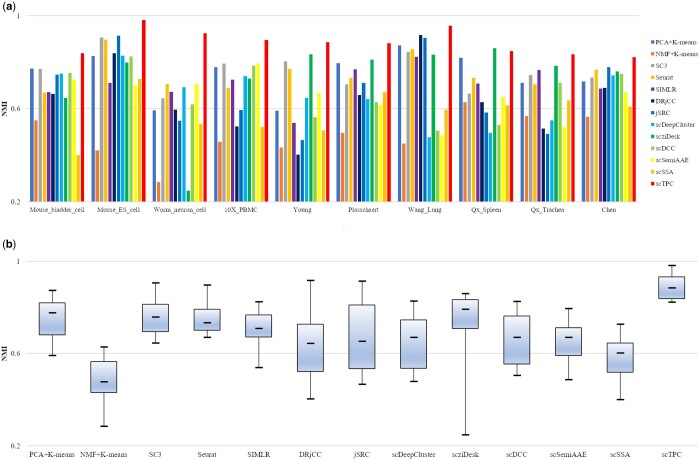
Clustering performance (a) NMI and (b) boxplot of different algorithms on 10 scRNA-seq datasets.

For each method, we repeated the process 10 times in every dataset and recorded the averages as the final metrics. We used 10% of labeled cells to generate semisupervised constraints and constructed 10 000 triplets. The clustering performance metrics, measured by NMI, for all 13 algorithms on the 10 datasets are visualized in Fig. 3 (ARI, ACC, and AMI can be found in the Supplementary File). It is evident that scTPC achieves good clustering performance regardless of the number of cells in the dataset. Although it may not have the best performance in some individual datasets, it remains one of the top three. It should be noted that the clustering performance may not be good when the number of a single cell type is extremely limited. In the “Qx_Spleen” dataset, the “dendritic cell” category has significantly fewer samples compared to the other cell types. The extreme scarcity of a single cell type in the dataset could potentially contribute to the relatively inferior performance of scTPC.

To gain a better understanding of scTPC’s overall performance across all datasets, we created a boxplot that shows the clustering performance of the method in the 10 datasets. The results are presented in Fig. 3b. From the figure, it is evident that scTPC demonstrates the best overall performance and exhibits the most consistent results.

**Figure 4.**
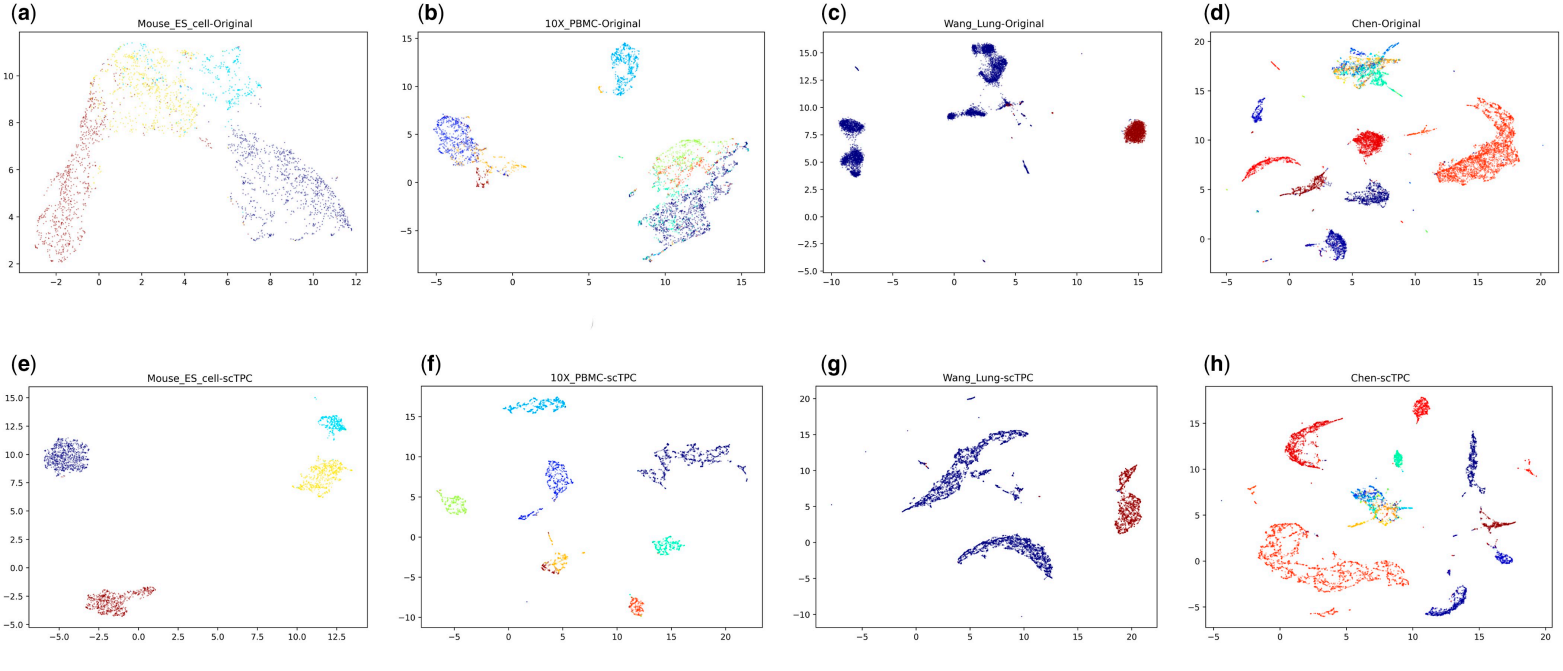
Visualizing clustering results on scRNA-seq datasets using UMAP. (a)–(d) are the visualization results of applying UMAP directly to datasets “Mouse_ES_cell,” “10X_PBMC,” “Wang_Lung,” and “Chen,” respectively. In contrast, (e)–(h) display the visualization results of the corresponding datasets after undergoing scTPC preprocessing.

The latent space referenced by the model represents an ideal low-dimensional embedding of the input high-dimensional data. To demonstrate the efficacy of the latent space, we employed UMAP (McInnes *et al.* 2018) to visualize the final embedding points obtained through model training in a 2D space. The results are presented in Fig. 4 (additional details can be found in the Supplementary File). In (a)–(d), we directly visualize the raw data, while (e)–(h) display the transformed data after model processing. We observed that cells appeared disorganized in the original space, with different clustered cell types that were difficult to discern. However, after model processing, distinct boundaries emerged, separating most of the different cell types and facilitating their identification, as cells of the same type clustered together.

**Figure 5.**
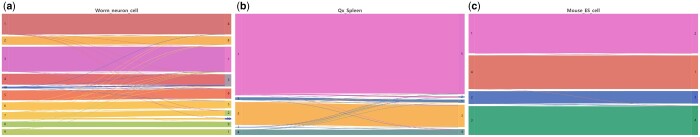
Sankey plots on the datasets. (a) “Worm_neuron_cell,” (b) “Qx_Spleen,” (c) “Mouse_ES_cell”.

Similarly, we illustrate the cluster assignment results for three datasets through Sankey diagrams in Fig. 5. The results for the other datasets are showed in the Supplementary File. Accurate clustering can be predominantly achieved when the dataset consists of a lesser number of cell types. However, in scenarios where numerous cell types exist, including some with limited representation, a degree of misassignment within cell clusters may occur. However, in general, our model demonstrates a relatively favorable clustering effect across diverse datasets.

### 3.4 Ablation studies and analysis

In this subsection, we delve into the rationale behind the introduction of several semisupervised constraints. To ensure a robust analysis, we maintain the unchanged structure of the remaining factors while adjusting one of them at a time. Through experimentation with authentic datasets, we successfully obtained the optimal model configuration.

**Figure 6.**
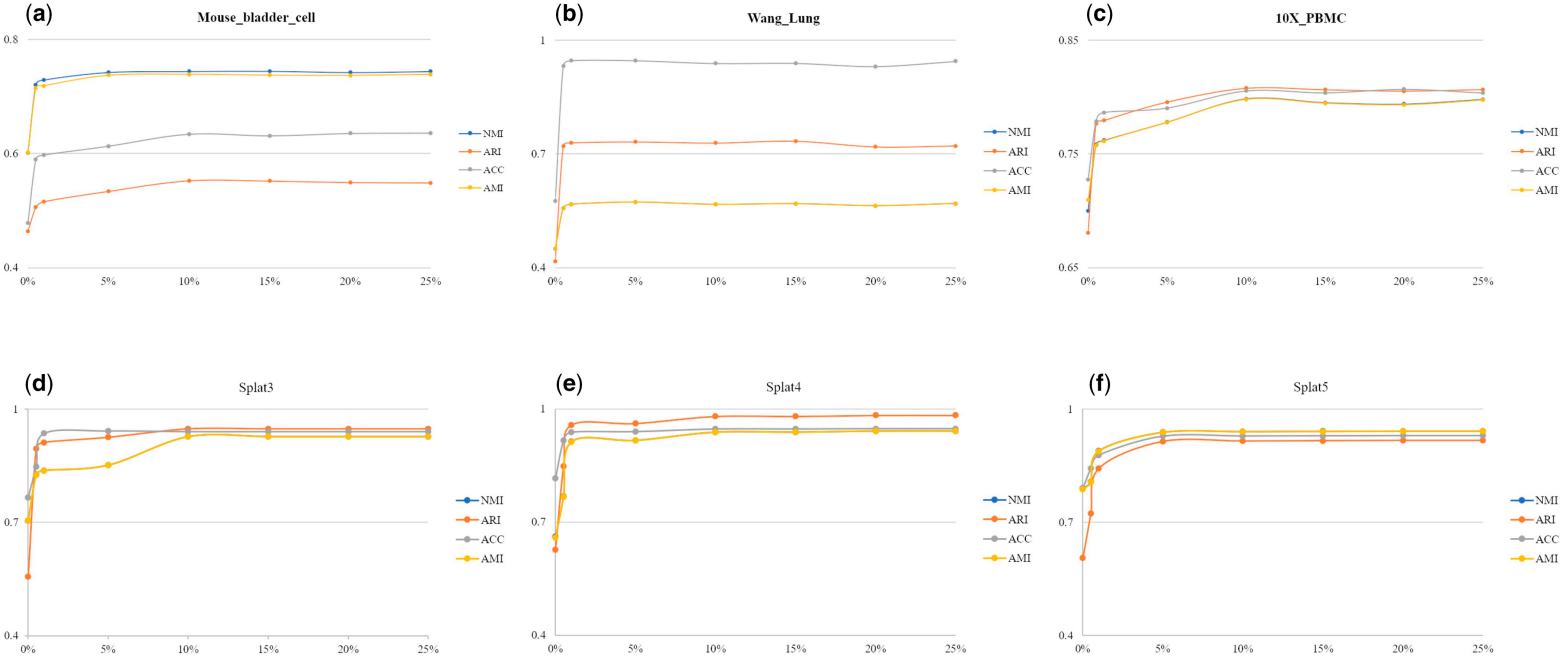
Clustering performance for the datasets with different proportion of labeled cells. (a) “Mouse_bladder_cell,” (b) “Wang_Lung,” (c) “10X_PBMC,” (d) “Splat3,” (e) “Splat4,” (f) “Splat5”.

Initially, the proportion of labeled cells is required. Thus, we set the proportions as 0%, 0.5%, 1%, 5%, 10%, 15%, 20%, and 25% and applied our method to investigate the clustering performance on real and simulated datasets. The results for datasets “Mouse_bladder_cell,” “Wang_Lung,” “10X_PBMC,” “Splat3,” “Splat4,” and “Splat5” are shown in Fig. 6 (the results for other datasets can be found in the Supplementary File). It can be observed that as the proportion of labeled cells used for constructing semisupervised constraints increases, the clustering performance improves. Furthermore, for most datasets, the clustering performance tends to plateau after reaching a proportion of labeled cells between 5% and 10%. As “Wang_Lung” only has two cell types, a small amount of labeled information yields favorable good clustering results. To have a standardized metric and ensure the sufficient triplets, we set 10% as the proportion of labeled cells for our proposed model.

**Figure 7.**
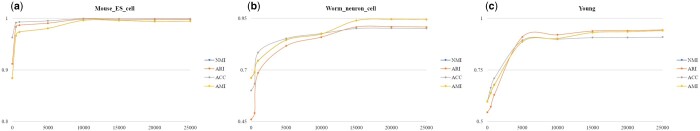
Determination of the number of triplets. (a) “Mouse_ES_cell,” (b) “Worm_neuron_cell,” (c) “Young”.

Next, in each dataset, we sought to determine the number of triplets needed to achieve optimal clustering performance using 10% of the labeled cells. We set the number of triplets as 0, 500, 1000, 5000, 10 000, 15 000, 20 000, and 25 000, and ran our method on the real datasets. The results for datasets “Mouse_ES_cell,” “Worm_neuron_cell,” and “Young” are shown in Fig. 7 (the results for the other datasets can be found in the Supplementary File). Notably, the number of triplets increases, the clustering performance gradually improves. For small-scale datasets, constructing 15 000 or more triplets leads to a plateau in clustering performance, whereas datasets with a larger number of cells require only 5000 triplets. Similarly, considering uniformity and computational cost, we set 10 000 as the number of triplets needed for optimal clustering performance.

**Figure 8.**
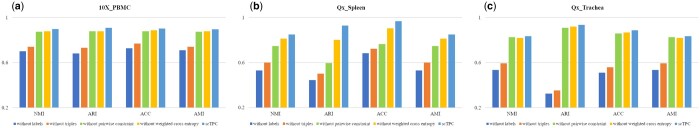
The impact of the introduced semisupervised constraints on the model. (a) “10X_PBMC,” (b) “Qx_Spleen,” and (c) “Qx_Trachea”.

To further investigate the impact of the introduced semisupervised constraints on the model, we conducted five groups of experiments for each dataset. These groups include: no labeled information, no triplet constraints, no pairwise constraints, no cross-entropy loss, and the complete model. We ran our model on real datasets, and the results for datasets “10X_PBMC,” “Qx_Spleen,” and “Qx_Tracheas” are shown in Fig. 8 (the results for other datasets can be found in the Supplementary File). Notably, when there is no labeled information, i.e. unsupervised clustering, the clustering performance is relatively poor and significantly lower than the results obtained by the complete model. Removal of the semisupervised constraints leads to a decrease in the clustering performance. In particular, the presence or absence of a triplet constraint causes significant differences in clustering performance, indicating that a triplet constraint contributes more to the model than the other two constraints.

## 4 Conclusion

The rapid development and widespread application of single-cell sequencing technology have provided researchers with a deeper understanding of transcriptomics. As the technology continues to advance, an increasing amount of scRNA-seq data can be obtained with biological prior information. Integrating prior information into clustering methods can assist researchers in conducting more in-depth single-cell studies. In this study, we investigated a deep learning-based, semisupervised clustering algorithm that incorporates prior information, which we refer to as scTPC. First, we constructed a denoising autoencoder to model the distribution of real scRNA-seq data using a zero-inflated negative binomial distribution and conducted pretraining. Through pretraining, we obtained the latent feature representation of the scRNA-seq data, and deep clustering was performed in a low-dimensional feature space. Triplet and pairwise constraints were generated based on known label cells, and latent expression and cluster centers were adjusted to obtain the final clustering results. Finally, considering the imbalanced nature of the cell categories in the dataset, we introduced a weighted cross-entropy optimization model.

To assess the performance of scTPC, we compared it with 12 other single-cell clustering methods in 10 real scRNA-seq datasets and five simulated datasets. The results consistently indicated that scTPC achieved favorable results in all datasets and exhibited superior clustering performance when considering the overall performance. To validate the rationale behind introducing constraints and optimize the model, we also conducted ablation experiments. These experiments helped determine the optimal number of triplet constraints and the ratio of pairwise constraints generated from labeled cells. Additionally, incorporating weighted cross-entropy to balance the dataset further enhanced the clustering performance. Overall, scTPC demonstrated its ability to accurately cluster scRNA-seq data, facilitating downstream analysis of sequencing data.

The method studied in this paper performs well on multiple datasets. However, due to the complexity of the model design, the training process incurs a high time cost. Therefore, future work will focus on optimizing the model structure to improve training efficiency. Additionally, based on the series of earlier experiments, we observed that the triplet constraint has a greater impact on the model than the other two constraints. Therefore, we will further explore these aspects in future research. Finally, with the availability of various omics sequencing data (Lin *et al.* 2022), we plan to apply the clustering framework to multiomics studies for more comprehensive analysis.

## Supplementary Material

btae293_Supplementary_Data
