# Supplementary material for: scTPC: a novel semisupervised deep clustering model for scRNA-seq data
Source: Bioinformatics. 2024 Apr 29;40(5):btae293. doi: 10.1093/bioinformatics/btae293 (PMC11091743; doi:10.1093/bioinformatics/btae293)
Supplement: btae293_Supplementary_Data [file btae293_supplementary_data.pdf]

Support materials

1 Support tables

1.1 Parameters

The parameters are determined through grid search, and the final results are as follows in the Table 1.

Table 1. Summary of the parameters.

Dataset	γ_1	γ_2	γ_3	γ_4
Mouse_bladder_cell	1	1	0.1	0.1
Mouse_ES_cell	0.1	0.1	0.05	0.05
Worm_neuron_cell	0.5	1	0.1	0.1
10X_PBMC	0.5	1	0.5	0.5
Young	0.1	1	0.05	0.05
Plasschaert	0.1	0.5	0.1	0.1
Wang_Lung	1	0.5	0.05	0.05
Qx_Spleen	0.1	0.5	0.1	0.5
Qx_Trachea	0.1	0.5	0.1	0.5
Chen	0.1	1	0.1	0.1

1.2 Compared methods

We compared to several advanced clustering methods, along with their corresponding programming languages and links, as shown in the Table 2.

Table 2. Summary of the compared methods.

Algorithm	Language	Link
SC3	R	https://github.com/hemberg-lab/SC3
Suerat	R	https://github.com/satijalab/seurat
SIMLR	R/Matlab	https://github.com/BatzoglouLabSU/SIMLR
DRjCC	Matlab	https://github.com/xkmaxidian/DRjCC
jSRC	Matlab	https://github.com/xkmaxidian/jSRC
scDeepCluster	Python	https://github.com/ttgump/scDeepCluster
scziDesk	Python	https://github.com/xuebaliang/scziDesk
scDCC	Python	https://github.com/ttgump/scDCC
scSSA	Python	https://github.com/houtongshuai123/scSSA/
scSemiAAE	Python	https://github.com/WHang98/scSemiAAE
PCA+K-means	Python	-
NMF+K-means	Python	-

2 Performance evaluation

Similar to most clustering methods, we select four commonly used metrics, namely NMI, AMI, ARI, and ACC, as the evaluation criteria.

2.1 Normalized Mutual Information (NMI)

The Mutual Information (MI) is defined as:

$$MI = \sum_x \sum_y p(x,y) \log \frac{p(x,y)}{p(x)p(y)}$$

Where $p(x,y)$ is the joint distribution of random variables X and Y, $p(x)$ and $p(y)$ are the marginal distributions of X and Y respectively, and NMI is defined as the mutual information between the predicted assignment U and the ground truth labels V divided by the entropy of U and V:

$$NMI = \frac{2MI}{H(U) + H(V)}$$

Where $H(U)$ and $H(V)$ represent the entropy of the predicted categories and the ground truth categories, respectively.

2.2 Adjusted Mutual Information (AMI)

The AMI measures the similarity between the clustering result and the ground truth labels, and is defined as:

$$AMI = \frac{MI - E[MI]}{\max(H(U), H(V)) - E[MI]}$$

Where MI represents mutual information,$E[MI]$ represents the expected mutual information, and $H(U)$ and $H(V)$ represent the entropy of the predicted categories and the ground truth categories, respectively.

2.3 Adjusted rand index (ARI)

ARI evaluates the similarity between predicted cluster assignments and true labels, and is defined as:

$$ARI = \frac{\binom{n}{2}(a+d) - [(a+b)(a+c) + (d+b)(d+c)]}{\binom{n}{2} - [(a+b)(a+c) + (d+b)(d+c)]}$$

Here, n represents the total number of cells; a represents the number of samples that are assigned to the same type in both the true cell types and clustering results; b represents the number of samples that are assigned to different cell types in the true cell types but the same type in the clustering results; c represents the number of samples that are assigned to the same type in the true cell types but different types in the clustering results; d represents the number of samples that are assigned to different cell types in both the true cell types and clustering results.

2.4 Clustering Accuracy (ACC)

ACC is defined as the best match between predicted cluster assignments and true labels:

$$ACC = \max_m \frac{\sum_{i=1}^{n} 1\{l_i = m(u_i)\}}{n}$$

Here, n represents the total number of cells; l_i represents the true labels; $m(\cdot)$ represents all possible one-to-one mappings between clustering assignments and true labels.

3 Support figures

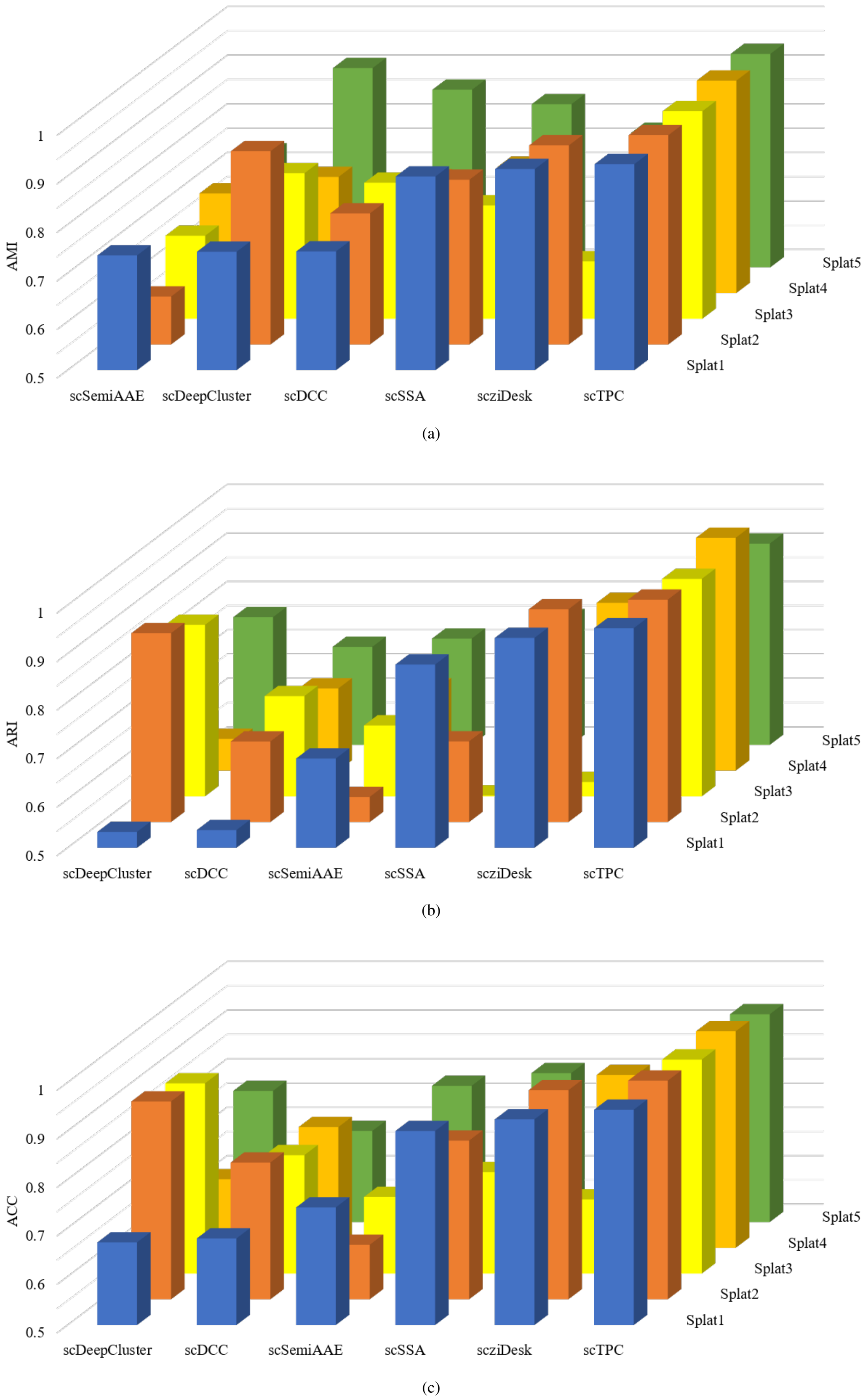

Fig. 1. Clustering performance of different algorithms on 5 simulated Datasets. (a) AMI , (b) ARI and (c) ACC.

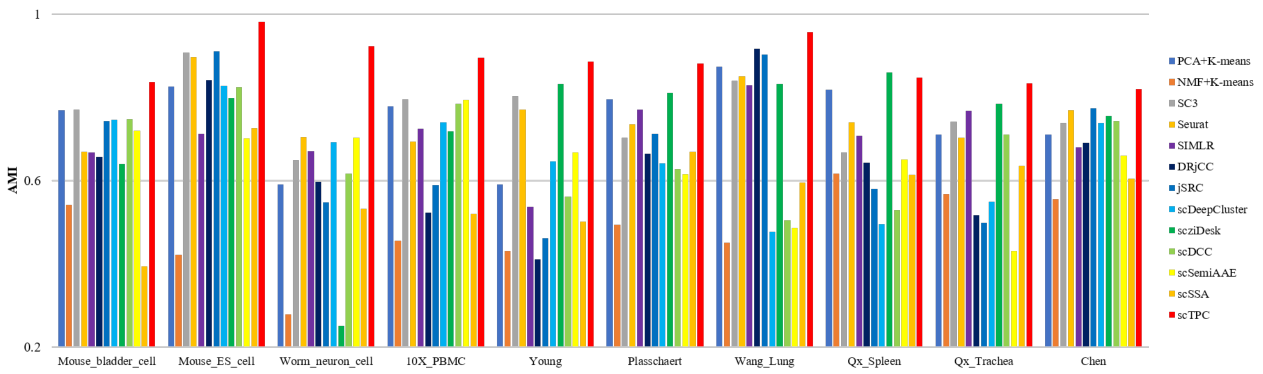

(a)

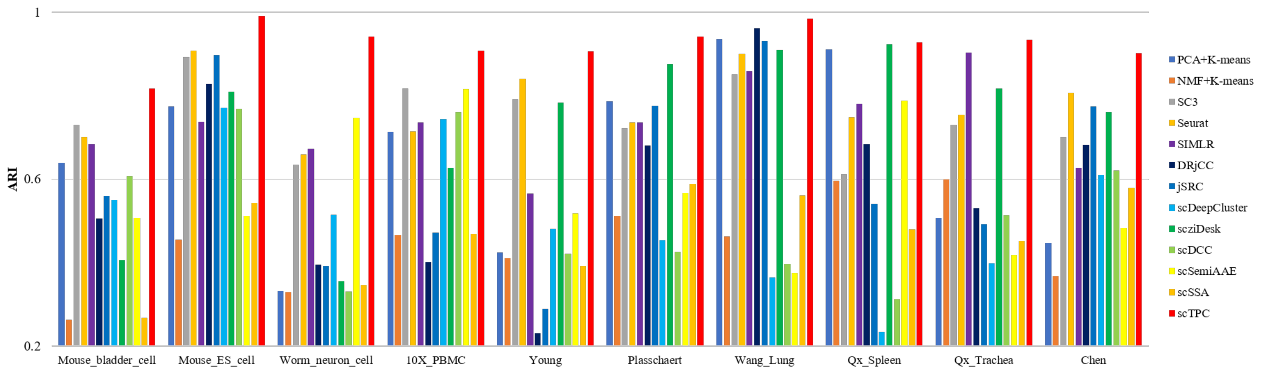

(b)

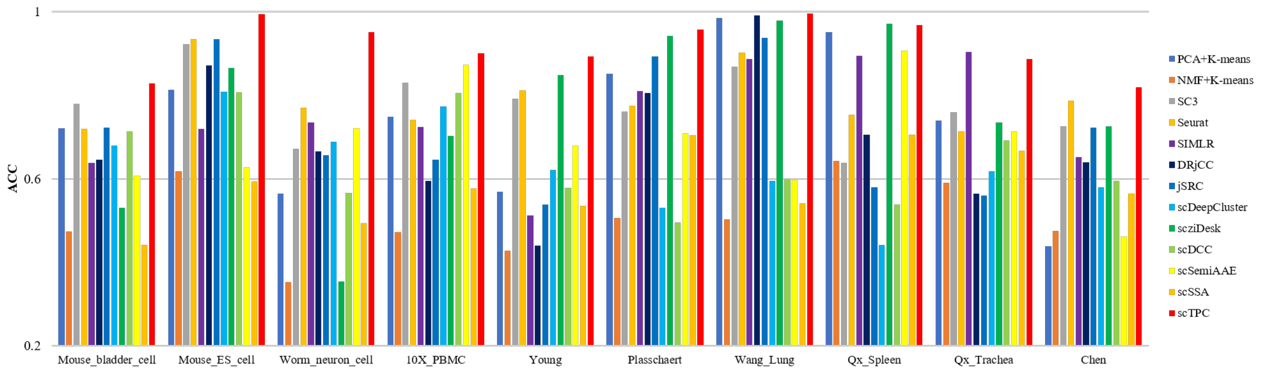

(c)

Fig. 2. Clustering performance of different algorithms on 10 scRNA-seq datasets. (a) AMI , (b) ARI and (c) ACC.

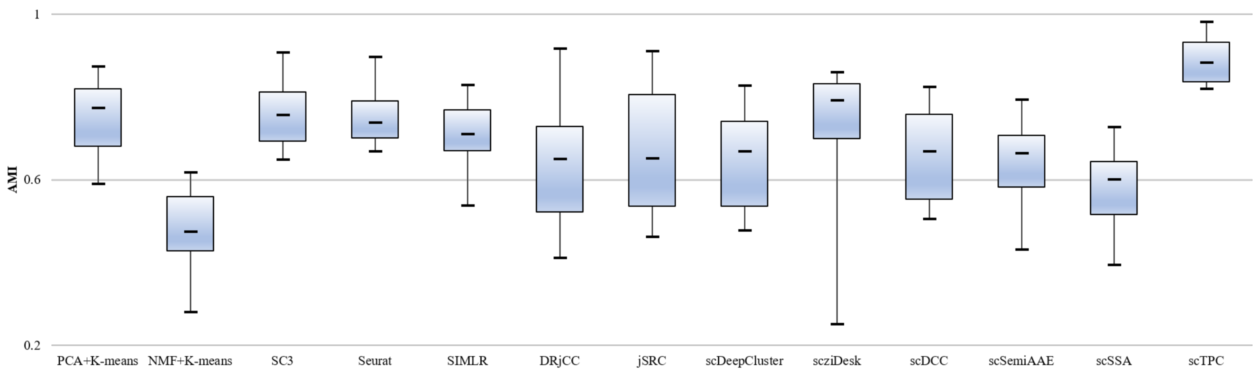

(a)

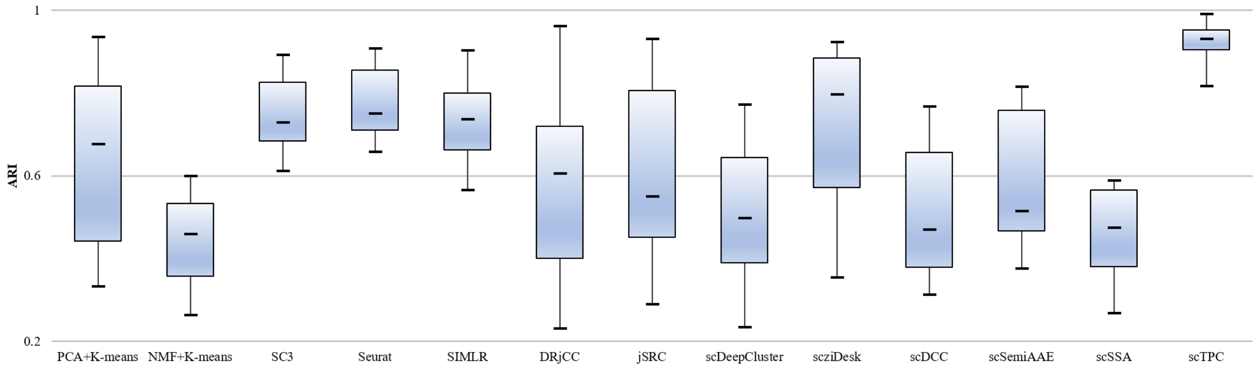

(b)

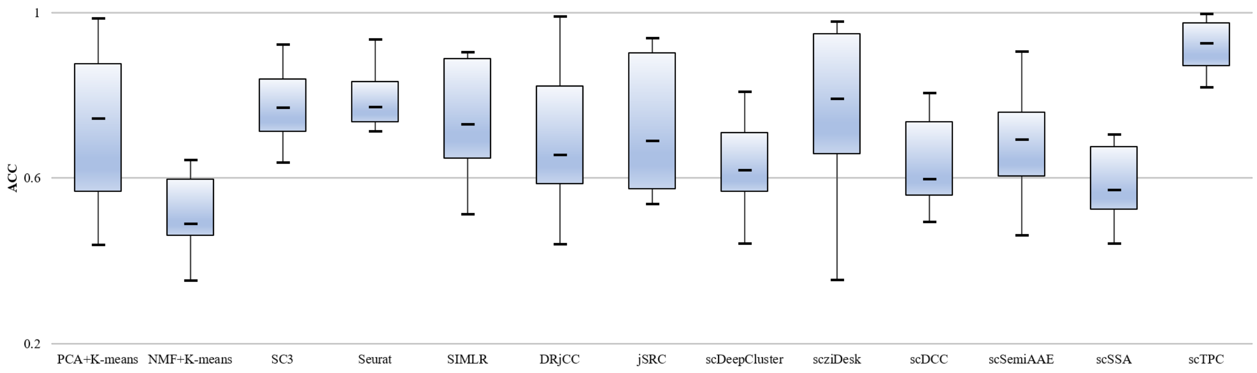

(c)

Fig. 3. Boxplot of different algorithms on 10 scRNA-seq datasets. (a) AMI , (b) ARI and (c) ACC.

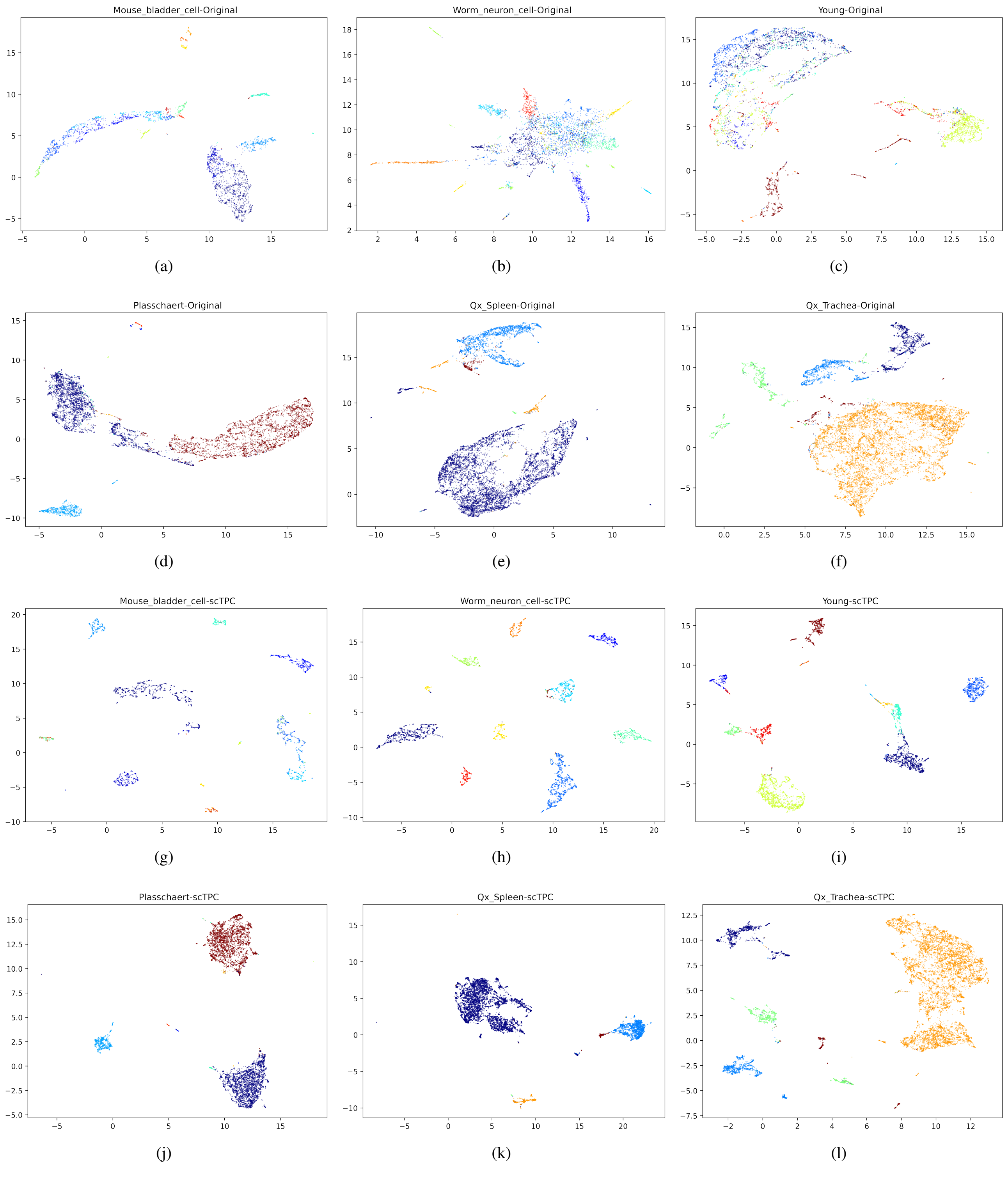

Fig. 4. UMAP visualization plots. (a)-(f) show the original data directly plotted. The other panels display visualization plots after processing with scTPC.

picture(0,0)(-35,0)(1,0)30 (0,35)(0,-1)30 picture picture(0,0)(35,0)(-1,0)30 (0,35)(0,-1)30 picture

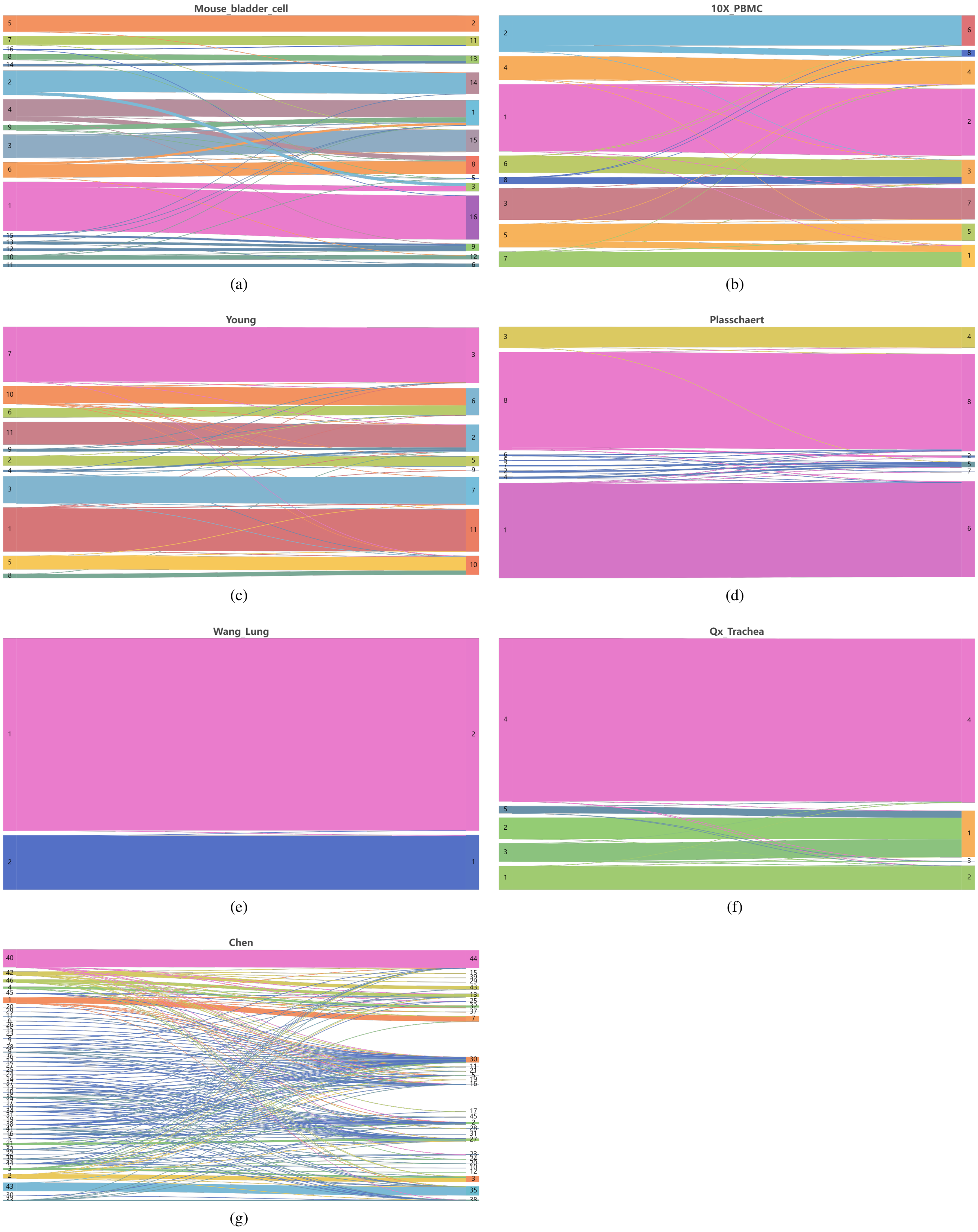

Fig. 5. Sankey plots.(a) "Mouse_bladder_cell", (b)"10X_PBMC", (c) "Young", (d) "Plasschaert", (e) "Wang_Lung", (f) "Qx_Trachea", (g) "Chen".

picture(0,0)(-35,0)(1,0)30 (0,-35)(0,1)30 picture picture(0,0)(35,0)(-1,0)30 (0,-35)(0,1)30 picture

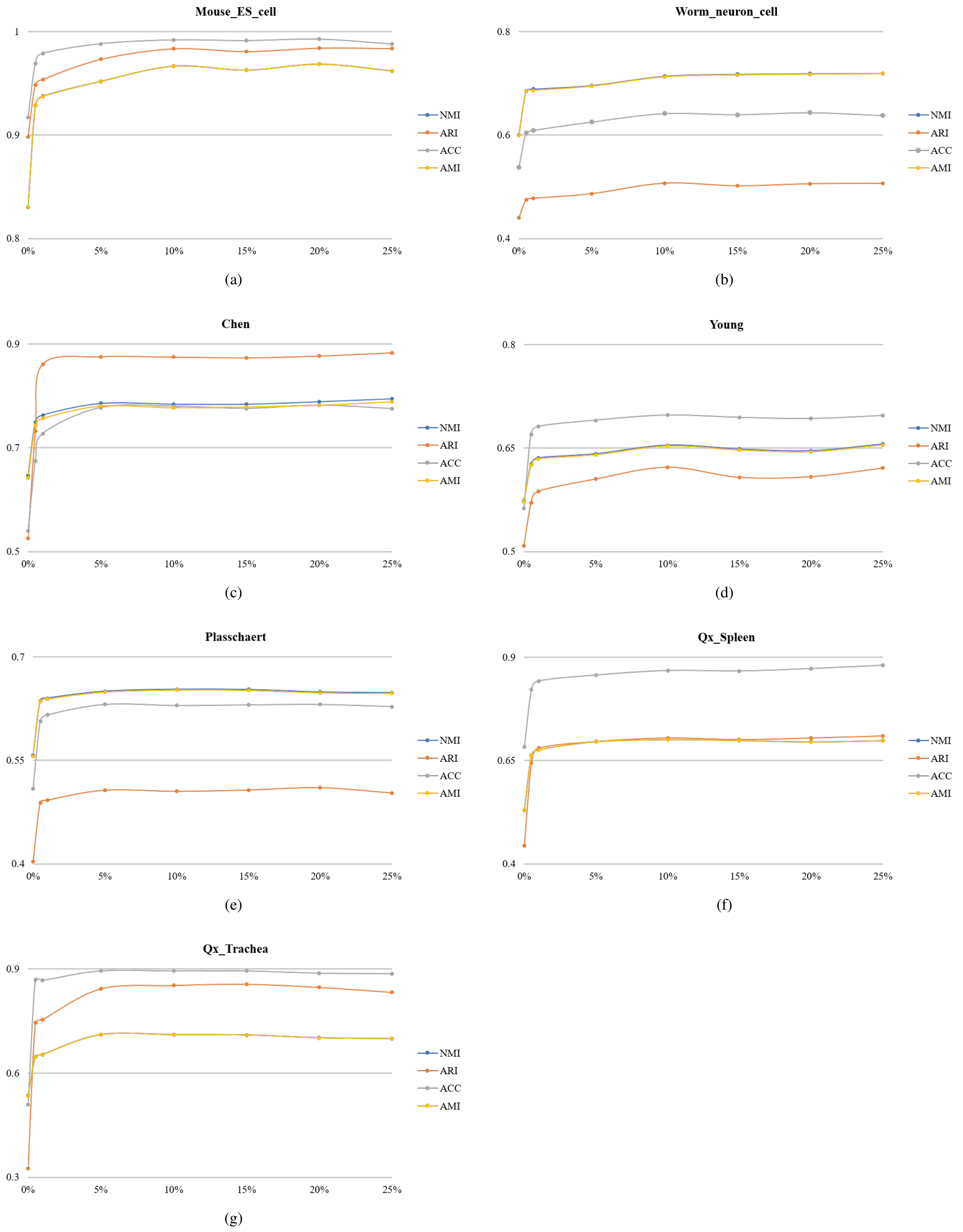

Fig. 6. Determination of the proportion of labeled cells.(a) "Mouse_ES_cell", (b) "Worm_neuron_cell", (c) "Chen", (d)"Young", (e)"Plasschaert", (f) "Qx_Spleen", (g) "Qx_Trachea".

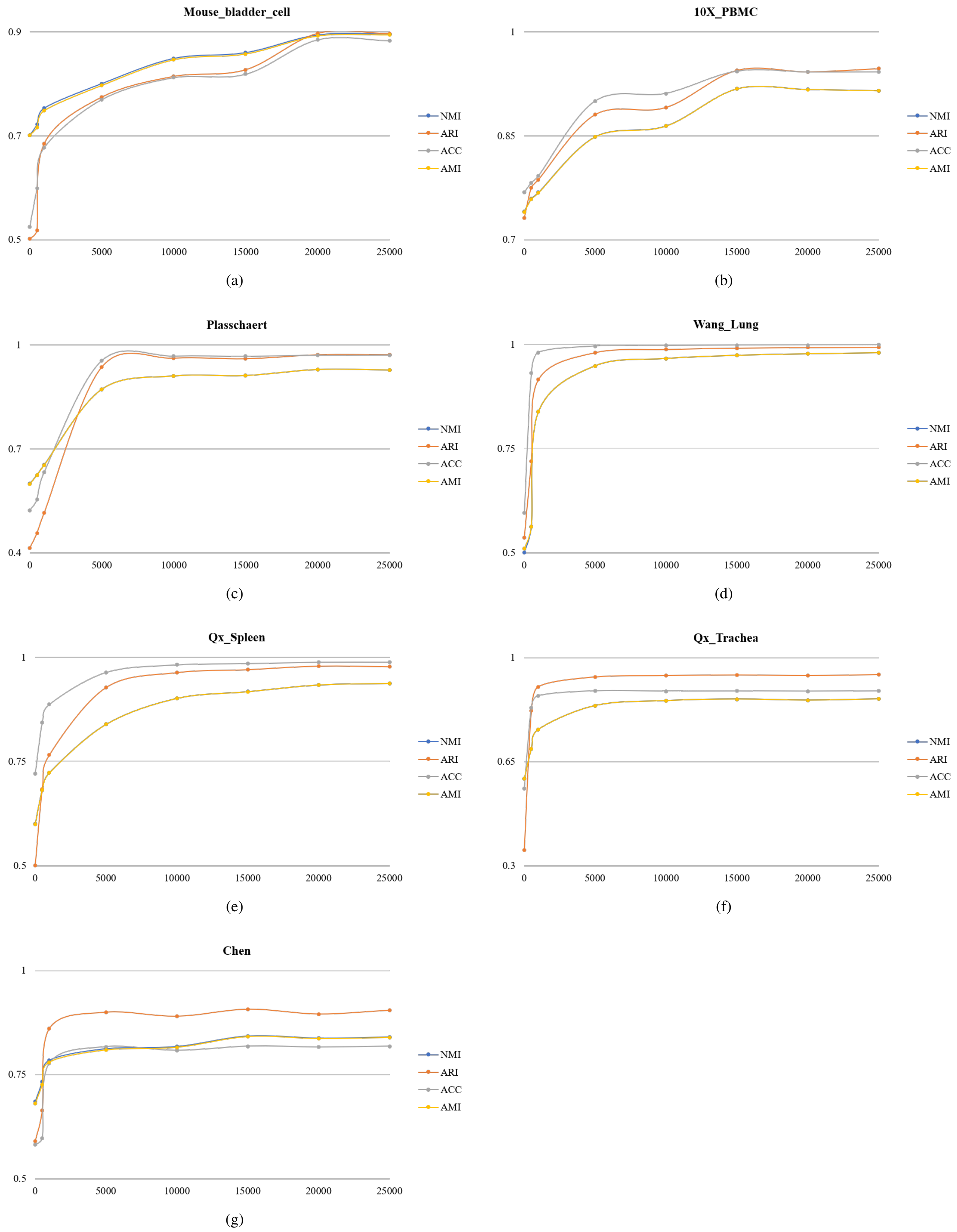

Fig. 7. Determination of the number of triples. (a) "Mouse_bladder_cell", (b)"10X_PBMC", (c) "Plasschaert", (d) "Wang_Lung", (e) "Qx_Spleen", (f) "Qx_Trachea", (g) "Chen".

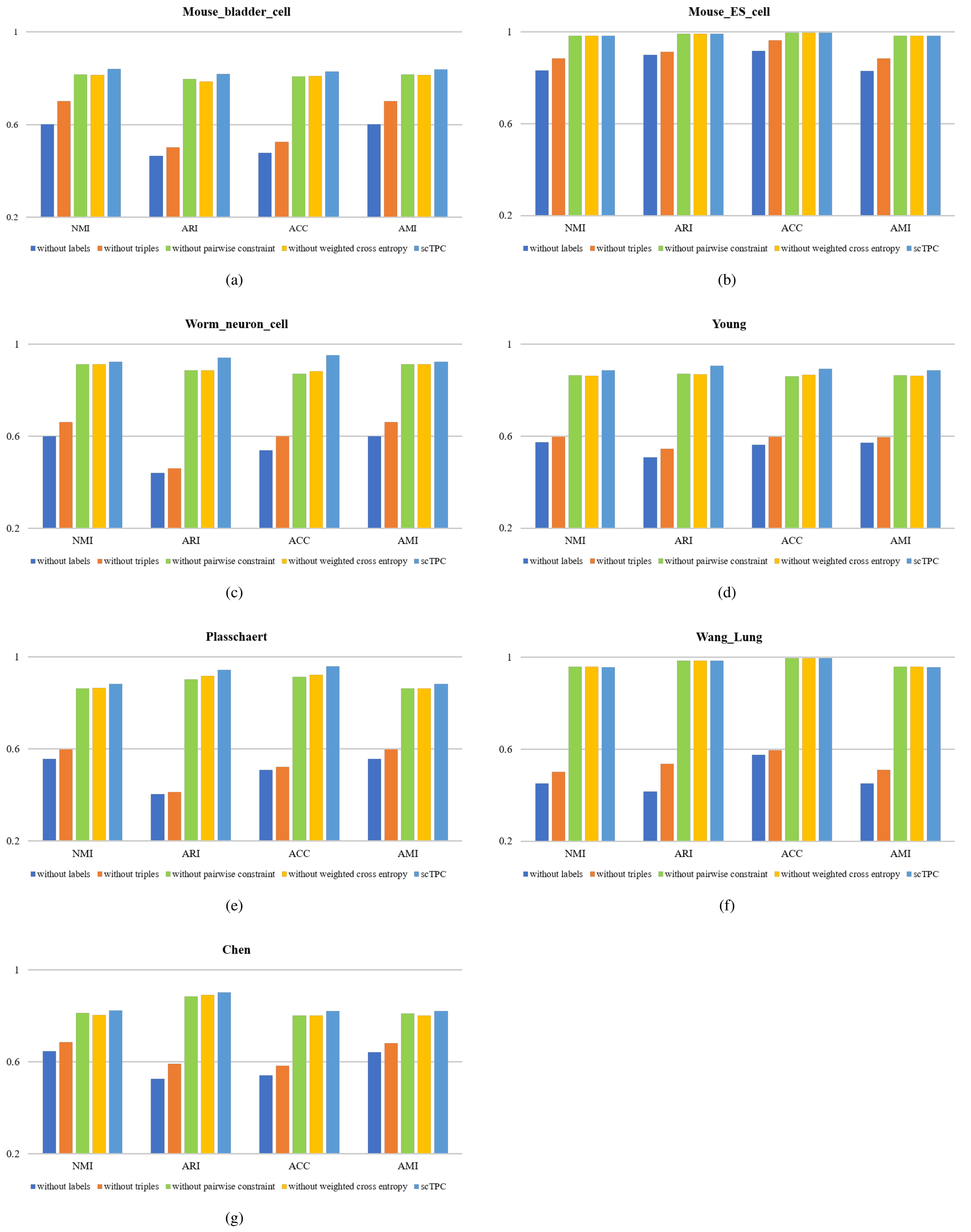

Fig. 8. The impact of the introduced semi-supervised constraints on the model. (a) "Mouse_bladder_cell", (b) "Mouse_ES_cell", (c) "Worm_neuron_cell", (d)"Young", (e)"Plasschaert", (f)"Wang_Lung", (g) "Chen".
